# Novel Therapies for Inherited Retinal Dystrophies

**DOI:** 10.3390/jcm13237358

**Published:** 2024-12-03

**Authors:** Christine Nichols Kay

**Affiliations:** Vitreoretinal Associates, Gainesville, FL 32607, USA; christine@vra-pa.com

## What Have We Learned in This Last Decade?

With the approval of the first retinal gene therapy, voretigene neparvovec [1], in December 2017 (for treatment of RPE65-associated retinal degeneration), the pathway was opened for other companies to develop investigational clinical trial programs for inherited retinal disease. Several programs have since progressed to Phase 3, including one trial currently in Phase 3 for treatment of RPGR-associated retinal degeneration (the LUMEOS study, identifier NCT04671433). Multiple programs have also shown auspicious starts in Phase 1/2 trials. Atsena Therapeutics released positive preliminary data from their first cohort on the treatment of X-linked retinoschisis (LIGHTHOUSE study, NCT05878860), citing schisis closure in two-thirds of patients and microperimetry improvements [2]. Atsena also recently published data on the positive safety/efficacy profile of their treatment in *Lancet* [3] citing clinically significant improvements in full-field stimulus testing and multi-luminance mobility testing in the high dose cohort. In July 2021, the FDA granted Alkeus Pharmaceuticals the breakthrough therapy designation for ALK-001 (C20-D3-vitamin A) for treatment of Stargardt disease (alkeuspharma.com), and recently, Ascidian Therapeutics announced the first ever IND clearance for an RNA exon editor and fast tracked ACDN-01 in Stargardt disease (ascidian-tx.com).

Approvable outcomes in the low vision space can be challenging, and work must be done with regulatory agencies to collaboratively seek endpoints that can effectively capture visual function or functional vision improvements in these patient populations. Promising therapies can fail to meet primary endpoints not necessarily due to the product failing to be safe or effective, but failure to statistically meet predetermined endpoints in rare disease populations. The question remains whether these limitations are inherent to the investigated product or perhaps due to the choice of endpoint and design of the trial to accurately understand the disease’s natural history and progression. Designing trials in the rare disease space is admittedly challenging, from patient recruitment to statistical considerations, as well as regulatory challenges with current approvable endpoints in these patient populations [4]. Perhaps due to an awareness of this inherent challenge, Peter Marks at the FDA has been supportive of approvals for rare orphan diseases; for example, the FDA is taking steps to help accelerate the development of novel drugs and biologics through the START program (Support for clinical Trials Advancing Rare disease Therapeutics Pilot Program) [5,6]. Hopefully the next few years will see additional FDA approvals in the rare retinal disease space as researchers and regulators seek to work collaboratively to identify safe and effective therapies and support them across the finish line.

Nonprofits in the US, such as the Foundation Fighting Blindness, have led collaborative efforts of uniting researchers, clinical MDs, and patient advocacy groups with regulatory leaders (FDA) to discuss improved understanding of and reach agreement on approval endpoints in these low-vision, rare disease populations [7]. Much work remains to be done, but the groundwork has been laid.

With the approval of our first and only retinal gene therapy recently behind us, post-commercialization data can and are being gathered, and additional learnings concerning the durability and long-term safety of subretinal gene therapy for IRD are being collected. Although some concerns about the development of chorioretinal (CR) atrophy in a minority of patients treated with Luxturna for RPE65-associated retinal dystrophy has been seen—and is being carefully evaluated and followed—patients presenting with atrophy appear to have excellent visual function with sustained improvements (visual acuity remained stable or improved in 83% patients and FST improved on average by 3-log units) [8]. Determining the reason behind the development of CR atrophy will be important as the field explores theories including mechanical stress from bleb creation, dose-related transgene overexpression, and host factors from stressed retina milieu. All four surgeons in a particular study investigating CR atrophy post-Luxturna administration injected the vector directly into the subretinal space without using saline pre-bleb, so the concentration of the vector (dose) as well as the ubiquitous CAG promoter have been mentioned as possible reasons for vector-related toxicity [8]. Learning which patients may be at risk for the development of atrophy will continue to be important, as will optimizing all potentially related factors (e.g., surgical delivery, dose, control of inflammation).

Optimizing surgical protocols has become a point of emphasis and attention, especially in the large retinal gene therapy space involving subretinal delivery, which is still the standard technique for gene therapy delivery for IRD programs due to the need to directly treat photoreceptors in most of these diseases. An understanding is growing from a vitreoretinal surgical perspective in optimal techniques for gentle bleb creation and propagation, intraoperative guided optical coherence tomography, and cannula considerations [9,10,11].

Gene-agnostic programs have also seen dramatic advancement in the past several years, with optogenetic programs achieving clinical progress in retinitis pigmentosa and Stargardt disease. Nanoscope Therapeutics targets remaining healthy bipolar cells with an intravitreally delivered AAV vector when photoreceptors have been extensively lost, and the preliminary efficacy results have been encouraging. The Phase 2b RESTORE randomized controlled clinical trial evaluating MCO-010, a mutation-agnostic gene therapy for patients with advanced retinitis pigmentosa (RP), met its primary endpoint, demonstrating a statistically significant improvement in best-corrected visual acuity (BCVA) at week 52 in both the high-dose (0.337 LogMAR; *p* = 0.021) and low-dose (0.382 LogMAR; *p* = 0.029) treatment groups compared to the sham control group (0.050 LogMAR) [12].

For the general ophthalmologist, the optometrist, or the retina specialist, it is our responsibility to stay up to date and be able to advise our IRD patients about updates in the ever-expanding landscape of clinical trials and novel therapies. AAO guidelines now recognize and recommend genetic testing for IRDs as a standard of care in the field [13,14]. Practitioners should counsel patients regarding the importance of obtaining a genetic diagnosis for their IRD or be able to refer patients to a center where genetic testing and counseling can be performed. Similarly, awareness of which potential clinical trials or novel therapies may be candidates for which IRD patients is an important part of the clinical care of these IRD patients.
